# Serum 25-hydroxyvitamin D levels and the risk of idiopathic central precocious puberty in girls

**DOI:** 10.1016/j.clinsp.2023.100244

**Published:** 2023-07-05

**Authors:** Dong-Mei Gan, Jie Fang, Ping-Ping Zhang, Yu-Dan Zhao, Ya-Nan Xu

**Affiliations:** Department of Pediatric Endocrinology, Ningbo Women and Children's Hospital, Ningbo, Zhejiang, China

**Keywords:** Idiopathic central precocious puberty, Serum 25-hydroxyvitamin D, Girls, Risk factors

## Abstract

**Introduction:**

Prior studies have found inconsistent results regarding the relationship between vitamin D status and Idiopathic Central Precocious Puberty (ICPP).

**Objective:**

To assess the role of serum 25-hydroxyvitamin D (25 [OH]D) levels in ICPP development.

**Method:**

The authors retrospectively collected data from 221 girls with ICPP and 144 healthy girls between January 2017 and December 2019. The participants’ serum 25(OH)D levels were measured using an automatic chemiluminescence method, and the association between serum 25(OH)D levels and the risk of ICPP was assessed using multivariate logistic regression analysis. Odds Ratios (OR) with 95% Confidence Intervals (95% CI) were calculated as effect estimates.

**Results:**

Serum 25(OH)D levels in the ICPP group were significantly lower than those in healthy controls (p < 0.001). Multivariate analysis indicated that girls with insufficient vitamin D levels (OR = 0.201; 95% CI 0.094–0.428; p < 0.001) and sufficient vitamin D levels (OR = 0.141; 95% CI 0.053–0.375; p < 0.001) both had a lower risk of ICPP than girls with vitamin D deficiency. Moreover, the authors found that the height (p = 0.014), weight (p = 0.014), breast stage (p = 0.010), mother's height (p < 0.001), and luteinizing hormone/follicle-stimulating hormone ratio (p = 0.010) in girls with ICPP could be associated with levels of vitamin D.

**Conclusion:**

This study found that a low serum 25(OH)D level is an independent risk factor for ICPP, and several characteristics of girls with ICPP could be affected by their vitamin D status.

## Highlights

• Insufficient vitamin D levels and sufficient vitamin D levels had a lower risk of ICPP.
• The vitamin D levels in ICPP girls could affect by height, weight, breast stage, mother's height, and luteinizing hormone/follicle-stimulating hormone ratio.
• The results could affect by the imbalance of girls in vitamin D deficiency, insufficiency, and sufficiency groups.

## Introduction

Central Precocious Puberty (CPP) was defined as gonadotropin-dependent or true precocious puberty with the development of sexual characteristics caused by the early reactivation of the Hypothalamic Pituitary Gonadal (HPG) axis in girls < 8 years.^1^^,^^2^ CPP occurs idiopathically in most girls affected with this condition; the children do not have congenital or acquired organic lesions in the central nervous system. Early activation of the HPG axis results in the development of signs of precocious puberty, including sex hormone secretion, gonadal and genital development, and secondary sexual characteristics. Early secretion of Estradiol (E2) increases the growth rate but promotes bone maturation, resulting in a short height in adulthood.^3^ Thus, independent risk factors for Idiopathic CPP (ICPP) in girls should be identified for early intervention to block the pituitary-ovarian axis.

ICPP pathogenesis is complex, involving both genetic and environmental factors.^4^ Vitamin D plays a key role in calcium and phosphorus metabolism, which can regulate bone metabolism.^5^^,^^6^ Moreover, vitamin D deficiency is significantly associated with obesity, metabolic syndrome, autoimmune diseases, insulin resistance, cardiovascular diseases, and cancer.^7^, ^8^, ^9^, ^10^ Prior studies have demonstrated that vitamin D levels are significantly associated with the onset of menarche,^11^, ^12^, ^13^, ^14^ while inconsistent results have been reported regarding the association of vitamin D with the development of ICPP. The authors, therefore, conducted this study to explore the role of serum 25-hydroxyvitamin D (25 [OH]D) concentrations in the development of ICPP in girls.

## Method

### Patients

A total of 221 girls with ICPP and 144 healthy girls were retrospectively recruited from the Ningbo Women and Children's Hospital between January 2017 and December 2019. This study was approved by the Institutional Review Board of the Ningbo Women and Children's Hospital ([2018] n° 26) and followed the STROBE Statement. All patients and their families were informed regarding the study, and written informed consent was obtained from all patients and their families. The definition of ICPP followed the 2015 guidelines of the Chinese Medical Association,^15^ which are based on: 1) the Development of secondary sex characteristics in girls < 8 years or menarche < 10 years; 2) Linear growth acceleration; 3) Bone age minus chronological age > 1 year; 4) Enlarged uterus, defined by at least one ovarian volume > 1 mL and more than one ovarian follicle diameter > 4 mm on ultrasound; and 5) HPG axis activation, as confirmed by peak Luteinizing Hormone (LH) response on the GnRH stimulation test, with peak LH level and LH/Follicle-Stimulating Hormone (FSH) ratio of ≥ 5 mIU/mL and > 0.6, respectively. Girls were excluded if they: 1) Had a history of defined etiology; 2) Received medications affecting the reproductive axis or used hormonal medications before diagnosis; 3) Had peripheral or organic causes of CPP; 4) Had tumors in the hypothalamus or pituitary gland; and 5) Had adrenal gland diseases or hypothyroidism.

### Serum 25(OH)D level

Girls were divided into the deficient, insufficient, and sufficient vitamin D groups, which were defined as serum 25(OH)D levels < 20 ng/mL, 20–30 ng/mL, and ≥30 ng/mL, respectively.^16^^,^^17^

### Data collection and measurement

A physical examination was conducted by a professionally trained pediatric endocrinologist. The heights and weights of the study participants were measured using standard methods similar to previous studies.^18^ Weight measurements were performed using a digital weighing scale (Shanghei Luheng Industry and Trade Co. Ltd.), and rounded to the nearest 100g, and height was measured using a sitting height meter (Shanghei Luheng Industry and Trade Co. Ltd.) and rounded to the nearest 0.1 cm. Body Mass Index (BMI) was calculated as weight/height 2 and was expressed as kg/m 2. Bone age was evaluated and confirmed by two pediatric endocrinologists based on left-hand radiographs according to Greulich and Pyle's standards.^19^ Fasting blood samples were obtained from the antecubital vein of the left elbow and allowed to settle at 4°C for 30 min. Plasma was isolated by centrifugation (2400 × g, 5 min). Serum LH, FSH, E2, and testosterone concentrations were measured using chemiluminescence (Access 2 Immunoassay System, Beckman Coulter, #81600N, USA). Serum Insulin-like Growth Factor 1 (IGF-1) levels were determined using chemiluminescence (IMMULITE 2000 Systems Analyzer, Siemens Diagnostic, Inc. Flanders, NJ, 07836, USA). Serum total cholesterol, triglyceride, high-density lipoprotein cholesterol, and low-density lipoprotein cholesterol levels were measured using standard enzymatic methods (Cobas 8000 modular analyzer series, Roche, Germany).

### Statistical analysis

The means (standard deviations) and events (frequencies) were used to describe continuous and categorical variables, respectively. Analysis of variance was performed to compare the differences in continuous variables among the three groups, while the differences among the groups for categorical variables were compared using the Kruskal-Wallis or Chi-Square test. The role of serum 25(OH)D levels in the development of ICPP was assessed by multivariate stepwise logistic regression, and the effect estimates were assessed using Odds Ratios (OR) with 95% Confidence Intervals (95% CI). All reported p-values were 2-sided, and the inspection level was 0.05. Statistical analysis was performed using SPSS software (version 22.0; Chicago, IL, United States).

## Results

### Characteristics of included girls

The baseline characteristics of the participants are summarized according to vitamin D status in Table 1. The height (p = 0.002), weight (p = 0.002), bone age (p < 0.001), bone age/age ratio (p < 0.001), 25(OH)D level (p < 0.001), and incidence of ICPP (p < 0.001) were statistically associated with the different groups. However, no significant differences among groups were observed for age (p = 0.551), BMI (p = 0.073), father's height (p = 0.754), or mother's height (p = 0.063).

**Table 1:** Clinical characteristics of girls according to vitamin D status.

		25(OH)D (ng/mL)			
Variables	Overall (n = 335)	Deficiency (n = 180)	Insufficiency (n = 102)	Sufficiency (n = 53)	p-value
Disease status (%)					<0.001
Healthy	114 (34.03)	31 (17.22)	54 (52.94)	29 (54.72)	
ICPP	221 (65.97)	149 (82.78)	48 (47.06)	24 (45.28)	
Age (years)	8.23±0.81	8.27±0.82	8.18±0.85	8.17±0.69	0.551
Height (cm)	132.13±6.40	133.21±6.25	130.51±6.58	131.61±5.90	0.002
Weight (kg)	29.37±4.78	30.18±4.83	28.09±4.60	29.09±4.50	0.002
BMI (kg/m ^2^)	16.75±1.90	16.94±1.98	16.41±1.73	16.73±1.86	0.073
Father's height (cm)	171.25±4.76	171.18±4.55	171.14±5.00	171.70±5.03	0.754
Mother's height (cm)	157.97±4.79	158.33±5.00	157.05±4.57	158.53±4.31	0.063
Bone age	9.34±1.38	9.64±1.36	8.99±1.33	8.96±1.29	<0.001
Bone age/age ratio	1.14±0.14	1.17±0.14	1.10±0.14	1.10±0.14	<0.001
25(OH)D (ng/mL)	21.01±7.79	15.91±4.76	22.82±1.82	34.81±3.84	<0.001

### Impact factors for ICPP

After adjusting for potential confounders, the authors noted that the insufficient (OR = 0.201; 95% CI 0.094–0.428; p < 0.001) and sufficient (OR = 0.141; 95% CI 0.053–0.375; p < 0.001) groups had a lower risk of ICPP than the deficient group. Moreover, a higher weight (OR = 1.097; 95% CI 1.010–1.193; p = 0.029) and bone age/age ratio (OR = 1.180; 95% CI 1.134–1.227; p < 0.001) were associated with an increased risk of ICPP (Table 2).

**Table 2:** Multivariate logistic regression for the risk of ICPP.

				OR and 95%CI			
Variable		β	OR	Lower	Upper	χ ^2^	p
25(OH)D (ng/mL)	Deficiency	Reference					
	Insufficiency	-1.605	0.201	0.094	0.428	17.324	<0.001
	Sufficiency	-1.963	0.141	0.053	0.375	15.372	<0.001
Weight (kg)		0.093	1.097	1.010	1.193	4.764	0.029
Bone age/age ratio		0.166	1.180	1.134	1.227	68.047	<0.001

### Characteristics of girls with ICPP

The clinical characteristics of the girls with ICPP according to vitamin D status are shown in Table 3. Overall, we found significant differences in height (p = 0.014), weight (p = 0.014), breast stage (p = 0.010), mother's height (p < 0.001), and LH/FSH ratio (p = 0.010) between groups. No other significant differences were observed between the groups.

**Table 3:** Clinical characteristics of ICPP girls according to vitamin D status.

	Vitamin D			
Variable	Deficiency (n = 149)	Insufficiency (n = 48)	Sufficiency (n = 24)	p-value
Age (years)	8.32±0.81	8.11±0.79	8.19±0.59	0.258
Height (cm)	134.00±6.11	131.29±5.93	134.69±4.64	0.014
Height-SDS	0.79±0.95	0.51±0.84	1.05±0.91	0.051
Weight (kg)	30.83±4.81	28.76±4.67	31.69±4.24	0.014
BMI (kg/m ^2^)	17.12±2.06	16.64±2.09	17.46±2.22	0.232
BMI-SDS	0.51±0.96	0.31±0.97	0.70±1.03	0.257
Breast (%)				0.010^a^
B2	75 (50.34)	37 (77.08)	14 (58.33)	
B3	63 (42.28)	8 (16.67)	9 (37.50)	
B4	11 (7.38)	3 (6.25)	1 (4.17)	
Father's height (cm)	171.26±4.61	170.31±5.22	172.58±4.54	0.156
Mother's height (cm)	158.55±5.09	155.62±3.60	159.46±3.64	<0.001
Bone age	9.95±1.19	9.74±1.09	9.81±0.88	0.476
Difference between bone age and age	1.64±0.95	1.62±0.96	1.62±0.77	0.994
Bone age/age ratio	1.20±0.12	1.20±0.13	1.20±0.10	0.970
LH	2.05±1.98	2.20±3.23	2.90±5.48	0.403
FSH	5.26±2.94	5.03±3.65	6.16±4.45	0.372
E2	25.91±29.79	18.43±24.42	25.62±24.09	0.271
IGF1	292.35±83.65	269.83±70.22	289.25±72.86	0.236
IGF1-SDS	0.19±0.95	0.09±0.91	0.26±0.99	0.736
FT3	4.41±0.75	4.52±0.86	4.45±0.58	0.657
FT4	1.03±1.12	1.32±2.44	0.95±0.13	0.450
TSH	3.37±2.47	3.40±1.65	3.82±2.11	0.666
T3	1.33±0.24	1.33±0.25	1.35±0.22	0.957
T4	8.51±6.71	10.43±15.65	8.14±1.60	0.413
ALP	342.60±79.16	313.27±73.54	341.79±78.64	0.074
Calcium	2.44±0.07	2.44±0.13	2.46±0.07	0.651
Phosphorus	1.75±0.14	1.79±0.25	1.79±0.12	0.338
Blood glucose	4.65±0.39	4.63±0.44	4.65±0.39	0.963
Uric acid	289.85±62.41	270.42±69.32	284.46±48.15	0.177
TG	0.96±0.40	0.97±0.31	0.88±0.29	0.619
TC	6.23±25.81	3.87±0.53	3.95±0.68	0.747
LDL	3.31±15.16	1.91±0.35	1.88±0.54	0.735
HDL	1.53±0.31	1.51±0.32	1.55±0.27	0.833
LH/FSH ratio	1.15±0.65	1.02±0.44	0.78±0.19	0.010

a ALP, Alkaline Phosphatase; BMI, Body Mass Index; E2 Estradiol; FSH, Follicle-Stimulating Hormone; FT, Free Triiodothyronine; HDL, High-Density Lipoprotein; IGF, Insulin-like Growth Factor; LDL, Low-Density Lipoprotein; LH, Luteinizing Hormone; SDS, SD Scores; T, Testosterone; TC, Total Cholesterol; TG, Triglyceride; TSH, Thyroid Stimulating Hormone.

## Discussion

Prior studies have demonstrated that early menarche is associated with an increased risk of obesity, type 2 diabetes mellitus, and cardiovascular disease.^20^, ^21^, ^22^, ^23^ However, the mechanisms underlying the early activation of the HPG axis remain unclear. The Vitamin D Receptor (VDR) is an intranuclear biological macromolecule that mediates the biological effects of vitamin D with biological activity; it is part of the steroid hormone receptor family. Vitamin D combines with VDR to exert hormone-like physiological effects. In the present retrospective study, the authors analyzed 221 ICPP patients and 144 healthy girls, with relatively good homogeneity in the characteristics of girls in both groups. Compared with the deficient group, the insufficient and sufficient groups were associated with a decreased risk of ICPP. Moreover, the authors found that the risk of ICPP may be affected by weight and bone age/age ratio. Finally, vitamin D levels were significantly associated with height, weight, breast stage, mother's height, and LH/FSH ratio in girls with ICPP.

Several studies have previously illustrated the role of vitamin D in ICPP.^24^, ^25^, ^26^ A prospective study conducted by Villamor et al. further found that vitamin D deficiency was significantly related to earlier menarche.^24^ Zhao et al. also found that vitamin D deficiency was significantly associated with an increased risk of ICPP, and a threshold effect of vitamin D status existed.^25^ Lee et al. found that the serum 25(OH)D level in girls with ICPP was significantly lower than that in healthy girls, and vitamin D deficiency was associated with an increased risk of ICPP.^26^ In the present study, the serum 25(OH)D level of girls with ICPP was significantly lower than that of healthy controls (18.56±6.66 vs. 25.15±7.84 ng/mL). Moreover, the authors noted that the majority of the identified girls presented with low serum 25(OH)D levels, which could be explained by a high school workload and reduced outdoor activities. Multivariate analysis revealed that the serum 25(OH)D level was an independent factor that could affect the risk of ICPP, which could be explained by the following: 1) Vitamin D deficiency was associated with an increased risk of adiposity, which could cause accelerated sexual maturation;^27^, ^28^, ^29^ 2) Serum 25(OH)D levels are inversely related to leptin concentrations, which could affect the progression of early puberty;^30^^,^^31^ and 3) IGF-I could stimulate the gonadotropin-releasing hormone pulse, thereby affecting the onset of puberty.^32^^,^^33^

When assessing the role of vitamin D status with the characteristics of girls with ICPP, the authors noted that height, weight, breast stage, mother's height, and LH/FSH ratio could all be affected by the vitamin D status, which is inconsistent with a previous study conducted by Bénazé et al.^34^ In this past study, the authors investigated 145 of 493 girls with ICPP, finding no significant differences between the serum 25(OH)D levels and puberty characteristics (BMI, growth rate the year before the onset of puberty, bone age, LH and FSH peaks, LH/FSH peak ratio, and E2 concentration).^34^ Height, weight, and breast stage were significantly associated with ICPP severity, which could be attributed to vitamin D levels. The difference in the mother's height among the groups was related to genetic factors and the severity of ICPP. Similarly, the authors found that the peak LH/FSH ratio can help distinguish rapidly progressive CPP from non-progressive CPP, with rapidly progressive CPP being significantly related to the LH/FSH ratio.^15^^,^^35^ Moreover, rapidly progressive CPP is associated with accelerated bone maturation and epiphyseal fusion. Finally, the authors found that estrogen can promote osteoblast differentiation and bone growth and promote osteoclast apoptosis, which promotes the conversion of 25(OH)D in the blood and leads to a further reduction of plasma 25(OH)D levels.

Several limitations of this study should be mentioned. First, selective or recall biases inherent to the retrospective nature of the present study could affect the reliability of the conclusions. Second, the number of girls in the vitamin D deficient, insufficient, and sufficient groups was not balanced, and the results were therefore not stable. Third, the severity of ICPP was not assessed, which may be related to vitamin D status, and could bias the potential role of vitamin D status in the risk or characteristics of ICPP. Finally, data on the participants’ background consumption of vitamin D or calcium from dietary supplements were not available and should be considered in further studies.

## Conclusion

In summary, the authors found that vitamin D deficiency is an independent risk factor for ICPP in girls. Moreover, the weight and bone age/age ratio could affect the progression of ICPP. Furthermore, the vitamin D status in girls with ICPP may affect several characteristics, including their height, weight, breast stage, and LH/FSH ratio. Further large-scale prospective studies should be performed to verify the findings of this study in girls with specific characteristics.

## List of abbreviations

ICPP, Idiopathic Central Precocious Puberty; 25 [OH]D, 25-Hydroxyvitamin D; CPP, Central Precocious Puberty; HPG, Hypothalamic-Pituitary-Gonadal; E2, Estradiol; LH, Luteinizing Hormone; FSH, Follicle-Stimulating Hormone; BMI, Body Mass Index; IGF-1, Insulin-like Growth Factor 1; OR, Odds Ratios; CI, Confidence Interval; VDR, Vitamin D Receptor.

## Authors contributions

All authors have read and approved the final version of this manuscript.

## Availability of data and materials

The datasets used and analyzed in the present study are available from the corresponding author upon reasonable request.

## Ethics approval and consent to participate

The authors can confirm that all methods were carried out in accordance with the ethical standards as laid down in the 1964 Declaration of Helsinki and its later amendments or comparable ethical standards. The authors confirm that written informed consent has been obtained from all participants from their parents and/or legal guardians. This study was approved by the Institutional Review Board of the Ningbo Women and Children's Hospital ([2018] n° 26) and followed the STROBE Statement.

## Consent for publication

Not applicable.

## CRediT authorship contribution statement

**Dong-Mei Gan:** Data curation, Writing – original draft, Visualization, Investigation. **Jie Fang:** Conceptualization, Methodology, Software, Data curation, Writing – original draft, Software, Validation, Writing – review & editing. **Ping-Ping Zhang:** Visualization, Investigation. **Yu-Dan Zhao:** Supervision. **Ya-Nan Xu:** Writing – review & editing.

## Conflicts of interest

The authors declare no conflicts of interest.
